# Deciphering Production Potential and Gonadal Differentiation of the Backcross Hybrids Between *Argopecten irradians* and *A. purpuratus*

**DOI:** 10.3390/ijms27167290

**Published:** 2026-08-15

**Authors:** Xiaotong Zhang, Bo Liu, Jinjing Wang, Fukai Wang, Junhao Ning, Yinchu Wang, Xinghai Zhu, Shaoxuan Wu, Chunde Wang

**Affiliations:** 1 College of Marine Science and Engineering, Qingdao Agricultural University, Qingdao 266109, China; xiaotong2020123@163.com (X.Z.); liubomusic@126.com (B.L.); wfk0618@163.com (F.W.); 2 Research and Development Center for Efficient Utilization of Coastal Bioresources, Yantai Institute of Coastal Zone Research, Chinese Academy of Sciences, Yantai 264003, China; wangjinjing2025@163.com (J.W.); jhning@yic.ac.cn (J.N.); ycwang@yic.ac.cn (Y.W.); 3 Yantai Key Laboratory of Scallop Breeding, Yantai 264003, China; 4 MOE Key Laboratory of Marine Genetics and Breeding, College of Marine Life Sciences, Ocean University of China, Qingdao 266003, China; xhzhu@ouc.edu.cn; 5 College of Fisheries, Ludong University, Yantai, 264025, China

**Keywords:** *Argopecten irradians*, *Argopecten purpuratus*, heterosis, genetic diversity, maternal parentage determination

## Abstract

The scallops of the genus *Argopecten* hold a significant position in the international shellfish aquaculture industry. However, due to their hermaphroditic nature, selfing reproduction has led to an increasingly severe phenomenon of germplasm degradation. The development of interspecific hybridization can effectively address this issue, but the fertility of interspecific hybrid offspring is greatly affected. Backcrossing appears to be a more practical and feasible solution, yet current research in this regard remains extremely scarce. In this study, the F1 generation of bay–Peruvian hybrid scallops (Aip derived from Ai ♀ × Ap ♂) and Peruvian–bay hybrid scallops (Api derived from Ap ♀ × Ai ♂) were successfully backcrossed with Peruvian scallops (*Argopecten purpuratus*, Ap) and bay scallops (*Argopecten irradians*, Ai) to produce F2 backcrossed population (Aipi derived from Aip ♀ × Ai ♂; Aipp derived from Aip ♀ × Ap ♂; Apii derived from Api ♀ × Ai ♂ and Apip derived from Api ♀ × Ap ♂). These F2 populations were comprehensively analyzed in terms of heterozygosity, gonadal development and differentiation, fertility, genetic diversity, and offspring identification. The results revealed that the fertilization and hatching rates of the F2 generation were significantly lower than those of the parental generations. However, they exhibited normal development through larval, juvenile, and adult stages, albeit with prolonged larval development periods. Notably, the juvenile and adult stages demonstrated significant hybrid vigor. The F2 generation retained hermaphroditic characteristics but also included individuals with exclusively female gonads. Cloning of *Dmrt1* and *Foxl2* genes in these scallops showed high similarity with the bay scallop’s corresponding genes, and *Dmrt1* is specifically expressed in the testis, while *Foxl2* is specifically expressed in the ovary, as is the case in the bay scallop. Eleven pairs of polymorphic loci were selected from developed microsatellite markers for bay scallops, and the analysis revealed that the polymorphism levels in the F2 backcrossed population were higher than those in purebred parental self-crosses. Additionally, the reliability of parental genetic information in the backcrossed population has also been corroborated according to the scallop descent identification experiments. The findings of this study enhance the understanding of Aipi, Aipp, Apii, and Apip, providing a theoretical foundation for the selection and breeding of scallop species with superior traits and addressing inbreeding decline in scallop culture.

## 1. Introduction

As the primary source of aquatic products, aquaculture output, predominantly mollusk production, reached 18.91 million tons in 2022, which accounted for 20.02% of the world’s total aquaculture output [1]. Bivalves are the main form of mollusks, in which *Argopecten* scallops are globally significant cultivated varieties [2]. In China, the northern subspecies of bay scallop (*Argopecten irradians*, Ai), introduced from the Atlantic coast of the United States in the early 1980 s, has become the primary economic shellfish in northern coastal regions due to the advantages of rapid growth, short breeding cycle, and broad temperature tolerance [3,4]. However, prolonged inbreeding and frequent self-fertilization have led to a severe problem of inbreeding depression in China’s bay scallop aquaculture, which seriously hampers the development of the scallop industry.

To avoid a series of problems caused by inbreeding depression, such as poor growth, germplasm degradation, and high mortality rates, Chinese aquatic breeding experts have undertaken extensive efforts, including genetic breeding programs and various restocking initiatives, achieving some success. Wang et al. (2008) introduced the Peruvian scallop (*Argopecten purpuratus*, Ap) from Peru. This medium-sized, fast-growing species can reach a shell height of up to 15 cm and has a lifespan of 7–10 years, being particularly noted for its large adductor muscle and excellent flavor [5,6]. To improve bay scallop germplasm, hybridization work of Ai and Ap was conducted. Compared with parental populations, F1 hybrids of Ai and Ap have shown significant advantages in growth, lifespan, resistance, and male sterility, indicating Ap carries superior genes that can enhance growth and resistance through interspecific hybridization [7]. Despite belonging to the same genus, reproductive isolation exists between Ai and Ap, leading to gamete infertility, especially male sterility, in F1 hybrid progeny, which can significantly reduce fertilization rate in the F2 hybrid progeny [8]. Backcrossing may be an effective method to address reproductive isolation in the process of fine hybrid strains construction. The mechanism by which backcrossing alleviates reproductive isolation and restores fertility is well established in classical genetics. By repeatedly crossing hybrid progeny back to one of the parental species, backcrossing progressively reduces the proportion of incompatible allelic combinations derived from the divergent parental genomes, while gradually restoring the genetic background of the recurrent parent. This process facilitates the recovery of normal meiotic chromosome pairing and gametogenesis, thereby ameliorating hybrid sterility. At the same time, backcrossing enables the introgression of desirable traits from the donor parent into the recurrent parent’s genetic background—a principle widely exploited in crop and livestock breeding. In Drosophila, pioneering studies by Noor et al. (2001) demonstrated that backcross hybrid males between *Drosophila pseudoobscura* and *D. persimilis* exhibit partial fertility recovery and can mediate gene flow between the two species, providing direct empirical evidence that backcrossing breaks down reproductive barriers [9].

Population genetic analysis and tracking key traits, like growth traits and gonad developmental status, is critical in the process of backcross strains construction. In recent years, molecular marker technology has been widely used in germplasm identification, variety improvement, genetic relationship analysis and phylogeny. Simple Sequence Repeats (SSRs) and mitochondrial DNA analysis are commonly used molecular marker techniques in genetic research and both have unique advantages in resolving population genetic diversity, evolutionary relationships, population structure and genetic disease mechanisms. SSRs are markers developed based on the polymorphism of short tandem repeats (e.g., 2–6 base repeats) in the genome, which are highly polymorphic, co-dominant and widely distributed. SSR can assess the level of genetic diversity of a species by detecting allele frequencies and heterozygosity within a population. Meanwhile, SSR can quantify genetic differentiation between different geographical groups, reveal gene flow and isolation mechanisms, and has also been used to identify the relationships of endangered species to help develop conservation strategies [10]. The mitochondrial genome is characterized by matrilineal inheritance, rapid evolutionary rate and conservative structure. The matrilineal genetic properties of mitochondrial DNA (mtDNA) make it an ideal marker for tracing the history of matrilineal migration and population expansion events. At the same time, conserved regions of mitochondrial genes (e.g., cytochrome oxidase Cytochrome c oxidase subunit I (*COI*), NADH dehydrogenase (*ND*) genes) are commonly used to construct evolutionary trees of species [11]. The combination of the two provides complementary information on the nuclear and mitochondrial genomes. For example, a Tarim *schizothoracine* study using both SSR and mitochondrial markers found that genetic diversity of nuclear genes (SSR) was lower than that of mitochondrial genes, suggesting that female dispersal may be limited [12]. This multi-marker strategy is particularly important in conservation biology and evolutionary studies.

In the previous study, hybrid strains of Ai and Ap were produced and displayed obvious growth vigor [6,7]. Regrettably, gamete infertility of F1 hybrid progeny seriously influenced the fertilization rate of F2 hybrid progeny. To explore more possibilities for germplasm improvement, in this study, the F2 backcross population was created by backcrossing the F1 population with Ai and Ap, respectively, to breed the F2 hybrid populations. Growth traits and gonad development status were monitored during the growth stages, compared with the parent scallops. Meanwhile, key genes associated with gonadal differentiation were studied and validated in the hybrid progeny. Subsequently, genetic analysis of backcrossed progeny and parental population was conducted based on 11 SSR loci with high polymorphism and specificity to calculate the polymorphism, genetic distance, and genetic similarity. Finally, to ensure the parentage of progeny, mtDNA was utilized for its unique matrilineal inheritance characteristics. The present study contributed to an in-depth understanding of the genetic basis of hybrid scallop strains and provided data support for scallop hybrid breeding work.

## 2. Results

### 2.1. Embryonic and Larval Development of Backcross Progeny

**Figure 1:**
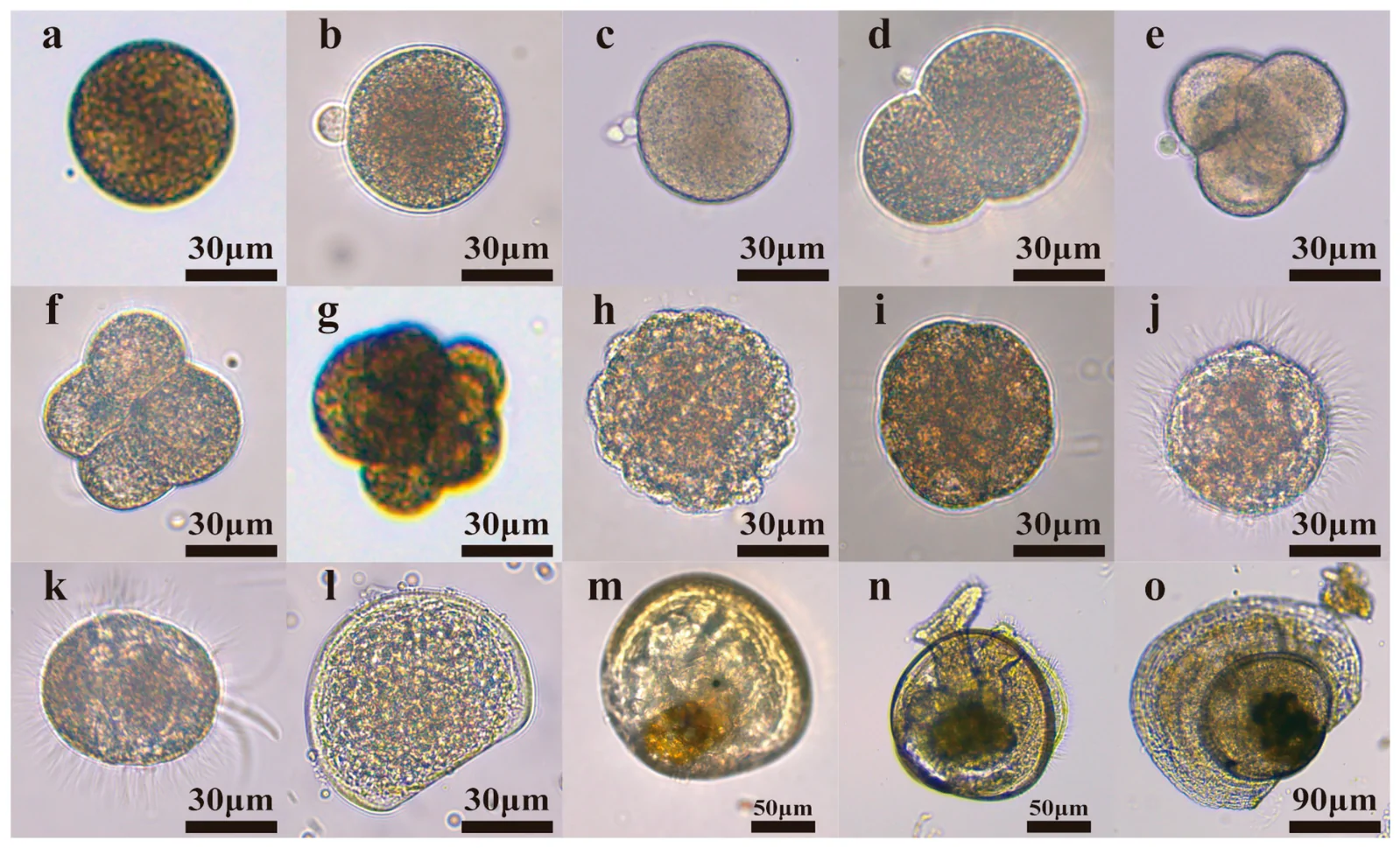
Embryonic and larval development of the purebred and backcross bred cohorts. (**a**): fertilized egg; (**b**): first polar body; (**c**): second polar body; (**d**): polar lobe; (**e**): two-cell stage; (**f**): four-cell stage; (**g**): eight-cell stage; (**h**): mulberry stage; (**i**): blastocyst stage; (**j**): gastrula stage; (**k**): trochophored; (**l**): D-larvae; (**m**): eyed larvae; (**n**): foot; (**o**): juvenile. Scale bars: (**a**–**l**) 30 µm; (**m**,**n**) 50 μm; (**o**) 90 µm.

**Figure 2:**
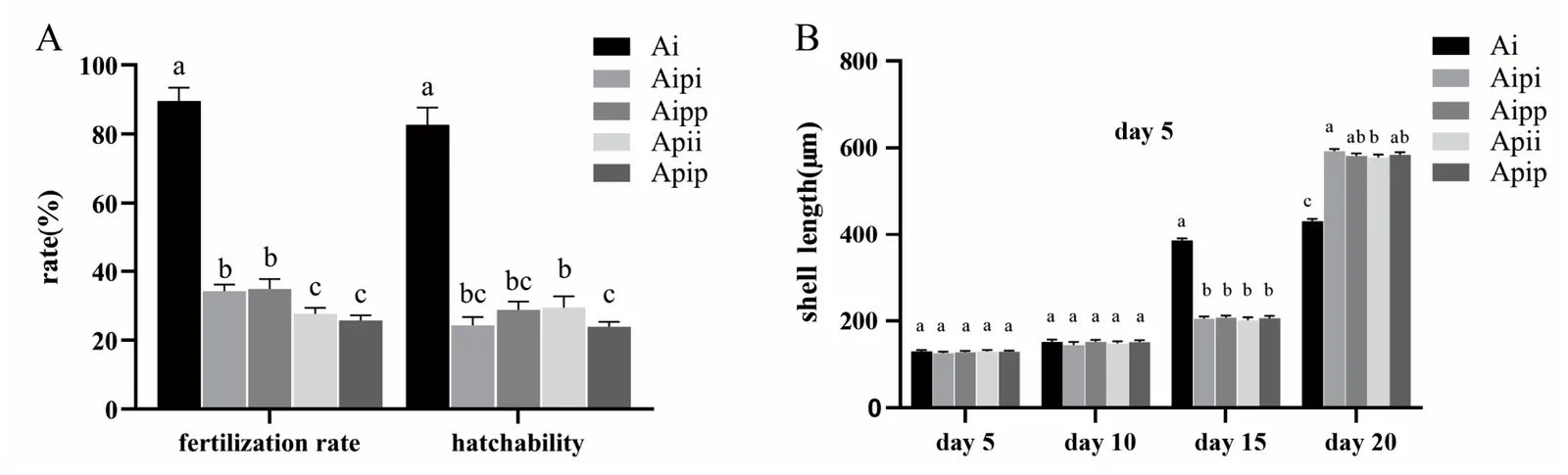
Reproductive and early growth statistics of Ai, Aipi, Aipp, Apii, and Apip. Values that lack a single conserved letter differ significantly (*p* < 0.05) from each other. (**A**): Fertilization and hatching rates. (**B**): Shell length statistics of larval and juvenile stage (*n* = 30).

Backcrossed hybrids were successfully obtained by hybridizing Aip/Api with Ap/Ai. The majority of embryos and larvae developed normally, showing that Aipi, Aipp, Apii, and Apip exhibited similar embryonic and larval developmental processes as Ai and Ap, progressing through fertilized egg, blastula, gastrula, trochophore larvae, D-larvae, eyespot larvae, settlement, and metamorphosis stages (Figure 1). Nevertheless, there were significant differences in fertilization and hatching rates between hybrids and parent individuals. As shown in Figure 2A, the fertilization rates of Ai, Aipi, Aipp, Apii, and Apip, determined at one hour post-insemination, were 89.65 ± 3.83%, 34.28 ± 1.94%, 34.96 ± 2.86%, 27.68 ± 1.77%, and 25.82 ± 1.44%, respectively. The fertilization rates of Aipi, Aipp, Apii, and Apip were significantly lower than that of Ai, with Apip showing the lowest rate. The hatching rates for Ai, Aipi, Aipp, Apii, and Apip were 82.69 ± 5.02%, 24.35 ± 2.41%, 28.87 ± 2.39%, 29.55 ± 3.21%, and 23.93 ± 1.43%, respectively. Similarly, the hatching rates of Aipi, Aipp, Apii, and Apip, measured 24 h post-fertilization when developing into D-larvae stage, were significantly lower than that of Ai.

Regarding developmental time, Aipi, Aipp, Apii, and Apip required longer periods to reach various stages of embryonic development compared to Ai. However, the differences in developmental time among the four hybrid groups were relatively minor (Table 1).

### 2.2. Statistical Analysis of Growth and Development

As shown in Figure 2B, during the larval period, growth was relatively smooth during the initial ten days, with Ai exhibiting a significantly accelerated growth rate compared to the other groups on day 15. By day 20, Aipi, Aipp, Apii, and Apip demonstrated a notable growth advantage compared with Ai, with no significant differences among these four groups.

The growth and development of Aipi, Aipp, Apii, and Apip were markedly superior to those of Ai (Table 2). Throughout the rearing stage, Aipi, Aipp, Apii, and Apip exhibited significant heterosis. When harvesting on the 150th day, the shell length dominance of Aipi, Aipp, Apii, and Apip was 5.48%, 27.98%, 22.75%, and 24.54%, respectively. Additionally, the wet weight advantages were 29.08%, 118.44%, 70.28%, and 99.50%, respectively. Among these groups, Aipi exhibited relatively low growth advantages.

### 2.3. Genetic Diversity Analysis

Genetic diversity was assessed using 11 microsatellite loci for Ai, Ap, Aipi, Aipp, Apii, and Apip. The backcrossed scallop populations displayed significant genetic stratification from the purebred populations. As shown in Table 3, the observed number of alleles (Na) per locus ranged from 1.2537 to 1.7164, while the effective number of alleles (Ne) varied between 1.2074 and 1.5295. Polymorphism levels in Aipi, Aipp, Apii, and Apip were significantly higher than those in the pure parental selfing lines. Additionally, Aipi, Aipp, Apii, and Apip exhibited elevated levels of polymorphism, as indicated by their higher percentages of polymorphic loci (P), Na, Ne, Nei’s gene diversity (H), and Shannon’s information index (I) compared to the pure parental self-crosses. Among these, Ap showed the lowest indices, whereas Apii demonstrated the highest level of genetic diversity. Analysis of genetic distance and genetic similarity revealed that Aipi and Apip, have high genetic similarity and small genetic distances with Ai (Table 4). Furthermore, Apii and Aipi exhibit a high degree of genetic similarity at the genotype level, whereas Ai and Ap show low genetic similarity and relatively long genetic distances.

### 2.4. Gonadal Development and Differentiation

**Figure 3:**
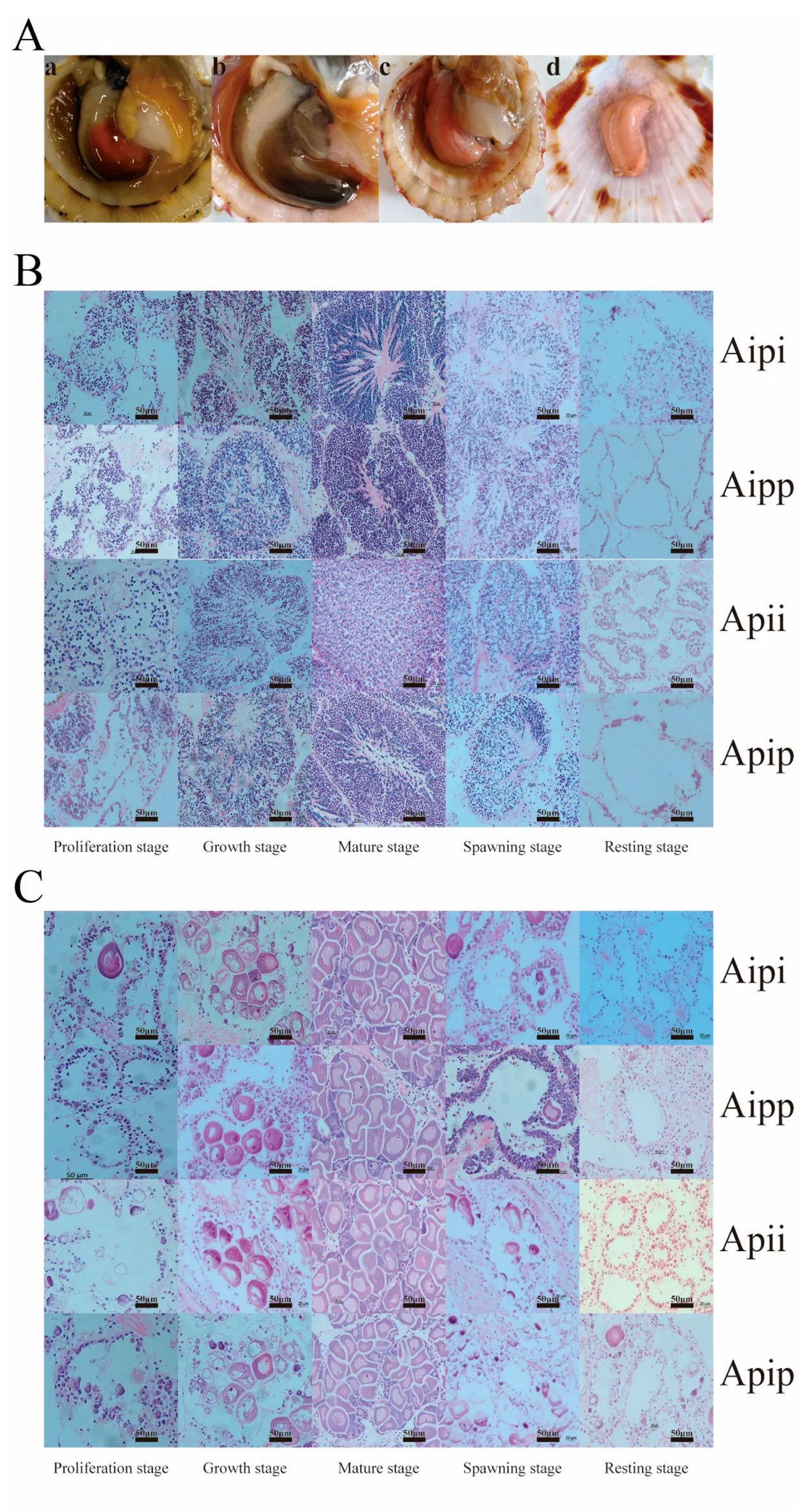
Sex phenotypes and gonadal development in Aipi, Aipp, Apii and Apip. (**A**): Phenotypic comparison between hermaphroditic and all-female individuals. The hermaphroditic gonads of purebred (**a**) and hybrid (**b**) scallops and the dioecious gonads in the backcrossed population (**c**,**d**). (**B**): Gonadal development of the spermatheca across five reproductive stages: proliferating stage, growth stage, maturing stage, spawning stage, and resting stage. A scale bar (50 μm) has been added to the bottom right corner of each image. (**C**): Gonadal development of the ovary across the same five reproductive stages: proliferating stage, growth stage, maturing stage, spawning stage, and resting stage. A scale bar (50 μm) has been added to the bottom right corner of each image.

Morphological and histological observations revealed that Aipi, Aipp, Apii, and Apip are hermaphroditic scallops, exhibiting normal development of spermathecae and ovaries with spermatozoa and egg production. However, spermatozoa activity was notably low. Additionally, Figure 3A shows the presence of individuals with all-female gonads among the adult scallops in the progeny of backcrossing populations. Unlike the hermaphroditic scallops, which exhibit distinct male and female reproductive zones, the all-female individuals displayed only ovarian tissue. Histological examination confirmed the absence of spermatozoa and spermatocytes in these individuals.

The development of the spermatheca and ovary followed distinct phases of proliferation, growth, maturation, discharge, and rest. These phases exhibited relatively similar developmental patterns among the groups during different periods of gonadal development (Figure 3B,C and Figure S1).

### 2.5. Gene Expression Related to Gonadal Differentiation

**Figure 4:**
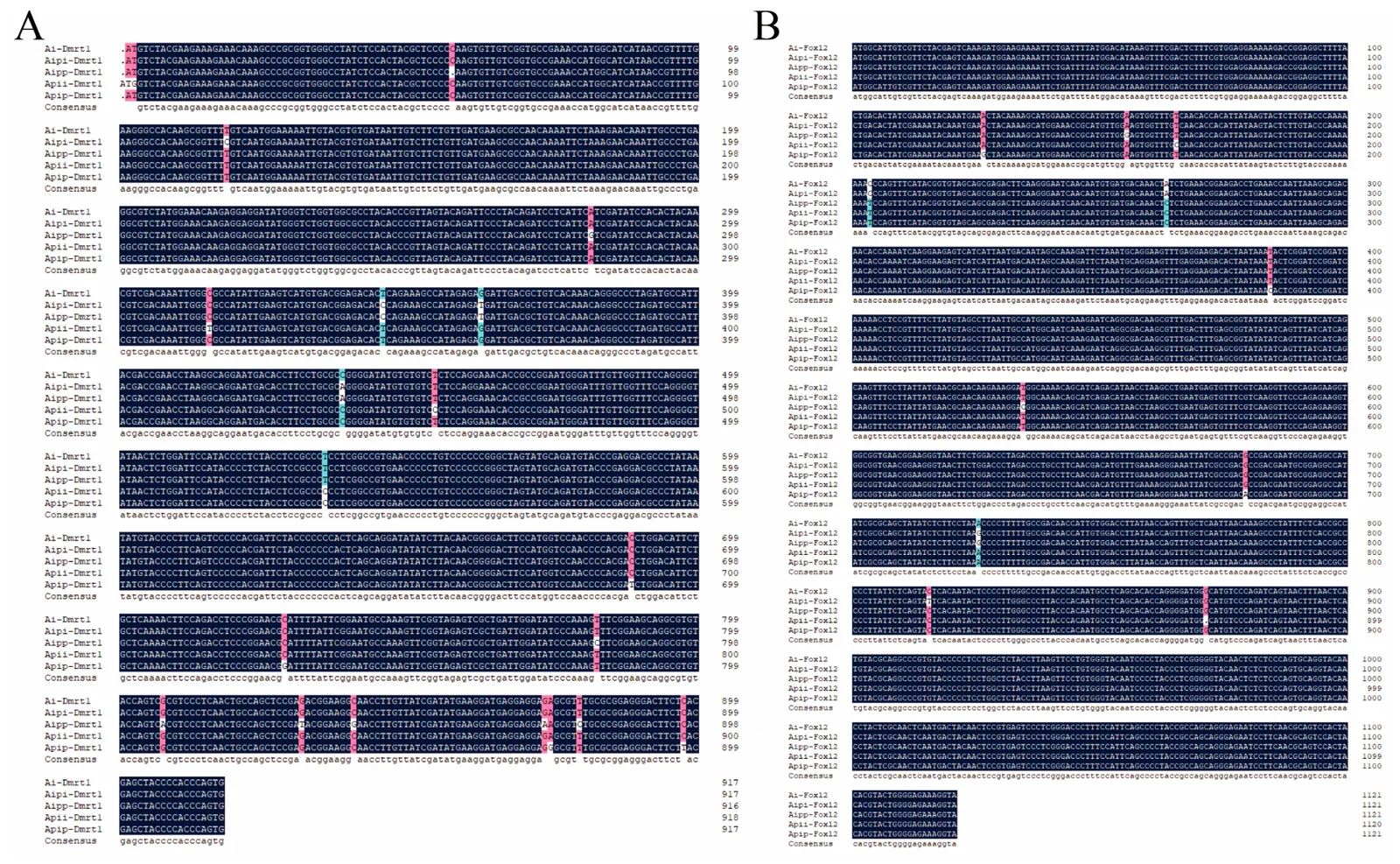
Sequence comparison of *Dmrt1* and *Foxl2* genes in Ai, Aipi, Aipp, Apii, and Apip scallops. The highlighted regions in the figure represent bases with sequence variations in the multiple-sequence alignment. (**A**): Sequence comparison of the *Dmrt1* gene. (**B**): Sequence comparison of the *Foxl2* gene.

The complementary DNAs (cDNAs) of four scallops, Aipi, Aipp, Apii, and Apip, served as templates for cloning and sequencing the *Dmrt1* and *Foxl2* genes. The resulting sequences underwent multiple sequence alignment with the *Dmrt1* and *Foxl2* genes of the bay scallop. The alignment results indicate that *Dmrt1* and *Foxl2* genes of individuals from each backcross population were separately 99.35% and 99.75% genetically similar to those in Ai, with differences due to single-base mutations (Figure 4A,B).

**Figure 5:**
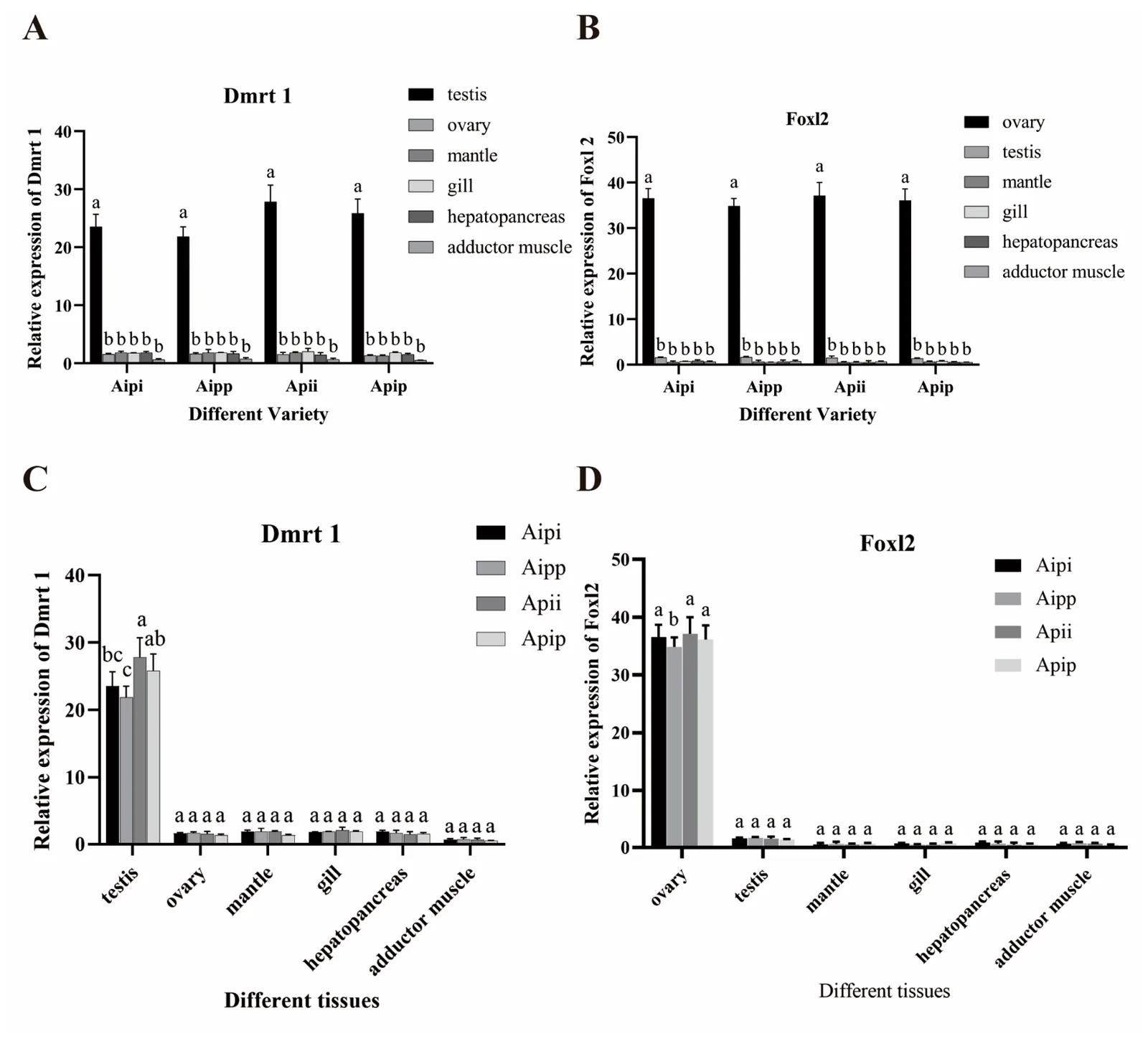
Tissue-specific expression analysis of *Dmrt1* and *Foxl2* genes in Aipi, Aipp, Apii, and Apip scallops. Values that lack a single conserved letter differ significantly (*p* < 0.05) from each other. (**A**): Tissue-specific expression of the *Dmrt1* gene in different variety. (**B**): Tissue-specific expression of the *Foxl2* gene in different variet. (**C**): Tissue-specific expression of the *Dmrt1* gene in different issue. (**D**): Tissue-specific expression of the *Foxl2* gene in different issue.

The quantitative real-time polymerase chain reaction (qRT-PCR) results revealed that *Dmrt1* was significantly more highly expressed in the testis than in all other tissues, with consistent expression levels observed in all varieties. In contrast, *Dmrt1* expression in the ovary, mantle, gill, hepatopancreas, and adductor muscle was consistently low and showed no statistically significant differences among them (Figure 5A). On the contrary, *Foxl2* was highly expressed in the ovary across all varieties, with consistent expression levels observed in all varieties. In contrast, its expression in other tissues was minimal, and no significant differences were observed among them (Figure 5B). To further characterize gonadal gene expression across the four backcross populations, we conducted inter-group statistical analyses of testicular *Dmrt1* and ovarian *Foxl2* expression. *Dmrt1* transcript abundance in testes peaked in Apii, followed by Apip, Aipp, and Aipi, which was likely attributable to substantial individual variation within each group (Figure 5C). Consistent with this pattern, ovarian *Foxl2* expression reached the maximum in Apii and the minimum in Aipi (Figure 5D). From the perspective of expression levels alone, the expression levels of *Dmrt1* and *Foxl2* in the gonads of Apii were the highest among all groups, although no significant differences were observed among the groups.

### 2.6. Results of Maternal Parentage Analyses

**Figure 6:**
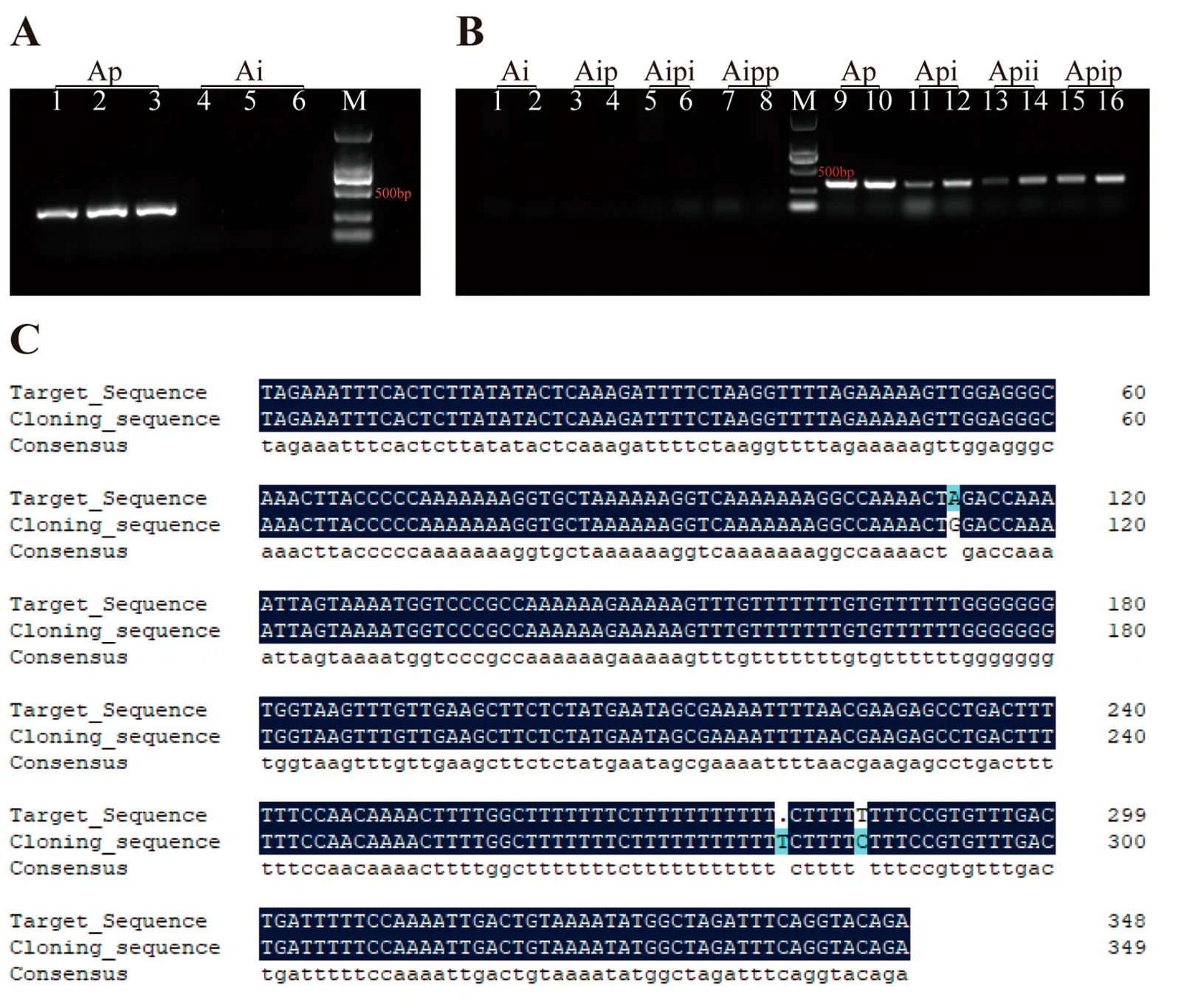
Electropherograms of amplification products using Specific primers and comparison of cloned sequences with mitochondrial DNA sequences of Ap scallop. (**A**): Electropherogram of amplification products of Ai and Ap scallops using Specific primers. 1, 2, 3 for Ap; 4, 5, 6 for Ai; M for DL2000 Marker. (**B**): Electropherogram of amplification products of Ai, Ap, Aip, Api, Aipi, Aipp, Apii, and Apip scallops using Specific primers. 1, 2 for Ai; 3, 4 for Aip; 5, 6 for Aipi; 7, 8 for Aipp; M for DL2000 Marker; 9, 10 for Ap; 11, 12 for Api; 13, 14 for Apii; 15, 16 for Apip. (**C**): Comparison of cloned sequences with mitochondrial DNA sequences of Ap scallops. The highlighted regions in the figure represent bases with sequence variations in the multiple-sequence alignment.

Ten molecular markers were designed over the mtDNA genomes of Ai and Ap (Table S1). During the screening stage, markers mbqq-2 through -10 failed to differentiate significantly between Ai and Ap. However, marker mbqq-1 successfully amplified a band of approximately 350 bp in Ap, with no amplification observed in Ai (Figure 6A and Figure S2A). The sizes of the target bands matched the significantly different sequences that had been screened, leading to the identification of marker mbqq-1 as a specific marker, subsequently designated as mbqq-Z.

The amplified product was sequenced, revealing a size of 348 bp. As shown in Figure 6B and Figure S1B, the sequence was compared with the Ap mtDNA sequence using DNAMAN for multiple sequence alignment. The clone sequence exhibited a 99.14% similarity with the Ap mtDNA sequence, confirming that primer mbqq-Z is amplified using Ap mtDNA as a template, ruling out interference from the nuclear genome sequence. This demonstrates that mbqq-Z is an appropriate specific primer for subsequent parentage identification experiments.

Finally, PCR amplification of Ai, Ap, Aip, Api, Aipi, Aipp, Apii, and Apip using primer mbqq-Z was performed, followed by detection using 1.2% agarose gel electrophoresis. The results, shown in Figure 6C, indicate successful amplification of a band of approximately 350 bp in Ap, Api, Apii, and Apip, with no significant amplification observed in Ai, Aip, Aipi, and Aipp.

## 3. Discussion

Inbreeding depression has long plagued scallop breeding work, and hybridization is an effective approach to mitigate this issue. In previous studies, hybrid breeding was conducted between Ai and Ap to improve the existing germplasm, and the research has firmly established the feasibility of crossing the scallop species Ai and Ap. However, the resulting progeny exhibits markedly reduced fertility due to the gametic isolation mechanism between Ai and Ap [7].

In the present study, a series of strains—Aipi, Aipp, Apii, and Apip—were obtained through the process of backcrossing the hybrid offspring Aip and Api with the parental species Ai and Ap, respectively. These strains exhibited lower fertilization and hatchability rates but were able to grow and develop normally during the embryonic and larval stages. Additionally, they demonstrated some heterosis. The gonads of the backcrossing population developed and matured normally, producing eggs as expected. In contrast, sperm production was essentially absent, and when present, sperm exhibited low vigor. The offspring demonstrated normal development and growth advantages following fertilization, displaying traits superior to those of the parents. However, the developmental periods of the backcross progeny were longer than those of the parental lines. These findings parallel those observed in interspecific crosses between Ai and Ap, indicating that hybridization affects the developmental rate of scallop larvae to a certain extent [13]. The initial size of D-larvae from the backcross progeny was not significantly different from that of the parental inbred lines. However, the backcross progeny exhibited significantly enhanced growth traits relative to the parental lines, indicating that interspecific hybridization has the potential to improve the overall performance of the population. Similar outcomes have been documented in the hybridization of scallops within the *Argopecten* genus [6,14]. For instance, the F1 generation resulting from the Ai and Ap cross exhibited significantly increased wet weight and adductor muscle weight, with the wet weight being approximately double that of the control population [7].

At the molecular level, hybridization does not create new genes but integrates single-copy genes from two parents into a single fertilized egg, producing heterosis through the additive effect of these genes. In production, crossbreeding transmits desirable traits such as growth, survival, and resistance from both parents to the offspring. Additionally, crossbreeding can result in novel traits not expressed by the parents [15,16,17,18,19]. This study revealed that, although the hybrid progeny did not exhibit significant advantages over the parents during the larval stage, they demonstrated notable growth advantages during the rearing stage. The 150-day-old hybrid progeny showed significantly superior characteristics compared to the parents, including shell length, shell width, shell height, wet weight, and adductor muscle weight. At harvest, the shell length advantages of Aipi, Aipp, Apii, and Apip over the bay scallop were 5.48%, 27.98%, 22.75%, and 24.54%, respectively, while the wet weight advantages over the bay scallop reached 29.08%, 118.44%, 70.28%, and 99.50%, respectively. These results are similar to or even surpass the intergeneric hybrids between Ai and Ap, where the progeny showed approximately a 90% increase in wet weight and a 150% increase in adductor muscle weight compared to their parents [7]. Ap and Ai belong to the same genus but different species. The wider genetic variation between the two species than between subspecies may underlie the significant improvement in aquatic animal production traits [20]. The notable heterosis of backcross progeny in this study may be attributed to the remote interspecific cross between the Ai and Ap. Meanwhile, the backcross progeny also exhibits closer genetic proximity to each other than to their original parents (Api and Aip). Accordingly, Aip and Api were backcrossed with Ai and Ap, respectively, to integrate the superior genes and traits of both species, reduce genetic distance and improve progeny fertility. This approach aims to provide a theoretical basis for the subsequent selection and breeding of scallop varieties with excellent traits and to address the serious problem of inbreeding decline in the scallop culture population.

The traditional crossbreeding of shellfish has been widely applied in marine shellfish aquaculture. Chinese scientists have utilized this technology to breed a novel species of scallops from the F1 hybrids between the Ap and Ai, including “Bohai Red”, “QN-2”, and “QN Orange” [21,22,23,24]. Nevertheless, the success of traditional crossbreeding depends on the successful combination of sperm and eggs between hybrid parents and the production of fertile offspring. Previous study indicated that gametic isolation mechanism between Ap and Ai constrained the fertilization and hatching rates of the hybrid offspring [7]. The results of this study, specifically manifested as male sterility and relatively poor egg viability in Aip and Api, further confirmed that backcross progeny still influenced by the reproductive isolation between grandparents. Meanwhile, individual dioecious hybrids with distinct female gonads were identified in the backcross progeny, showing the same all-female gonads as the F1 generation of Ai and Ap crosses [6]. This phenomenon further explains the lower fertility of progeny in backcross offspring due to the effects of testicular development inhibition.

The process of gonadal development can be divided into five stages: proliferation, growth, maturation, discharge, and resting, which are similar among scallops in different groups. To further explore the molecular regulatory mechanism of gonadal development, expression profiles of *Dmrt1* and *Foxl2* were analyzed. The *Dmrt1* gene plays a key role in regulating gonadal development, including germ cell proliferation, differentiation, migration, and various functions [25,26,27]. In ctenophore scallops (*Azumapecten farreri*) and Ezo scallops (*Mizuhopecten yessoensis*), the *Dmrt1* gene is expressed exclusively in the gonads, with significantly higher expression in the spermatheca than in the ovary, peaking during the growth and maturation period [28,29]. On the contrary, *Foxl2* gene is essential for female sex determination and the maintenance and differentiation of ovarian function [30,31,32,33,34]. *Dmrt1* showed significantly elevated expression in the testis, while its expression in other tissues, including the ovary, was minimal. In contrast, *Foxl2* was highly expressed in the ovary but nearly undetectable in the testis and other somatic tissues. The reciprocal expression patterns of these two genes are in agreement with previous findings in various vertebrates and invertebrates [35,36,37,38,39,40]. It suggested that *Dmrt1* and *Foxl2* may play crucial roles in sex differentiation and gonad development and also indicated a conserved mechanism of sex regulation involving *Dmrt1-Foxl2* antagonism, which has been found in other species [41,42,43,44]. The high expression of *Dmrt1* and *Foxl2* in the Apii also suggested that its gonads may have a better developmental status compared to other backcross populations, and thus this population may be more suitable for the subsequent breeding of hybrid strains.

Hybridization can enhance production traits by increasing the genetic diversity and heterozygosity of the hybrids, resulting in a hybrid advantage over the parents [45]. This study revealed that the genetic diversity of the backcross populations Aipi, Aipp, Apii, and Apip was significantly greater than those of purebred self-crosses. The greater genetic diversity often implies that populations have potential advantages in aspects such as growth, stress resistance, and survival. The increase in genetic diversity of hybrid progeny is influenced by the genetic distance between the parents [46]. In this study, backcross populations exhibited significant growth heterosis, which may stem from the relatively large genetic distance between the grandparental lines Ai and Ap. The application of SSR molecular marker technology allows for the calculation of genetic distance between species populations at the gene level. This technology can be used to predict the heterosis of hybrid progeny and to conduct expression quantitative trait locus (eQTL) analysis in conjunction with the phenotypic traits of hybrid progeny, facilitating an in-depth analysis of the potential mechanisms underlying heterosis. The hybrid populations in this study exhibited high homogeneity, with small genetic distances between the hybrids and the maternal lineage, likely a result of the maternal effect on hybrid progeny. This finding aligns with previous studies involving crosses between ctenophore scallops and Ezo scallops, which also reported a genetic bias towards the mother in hybrid progeny loci [47]. The inheritance of nuclear and cytoplasmic genetic material from both parents, with a stronger influence from the mother, may explain the observed bias in traits and loci towards the maternal parent. This insight provides a new direction for future crossbreeding efforts, suggesting that artificial selection of females could “customize” hybrid progeny with desirable traits.

Mitochondrial DNA sequences have become essential molecular markers in species identification, phylogeny, and kinship analysis [48,49,50,51]. Mitochondria, as the only extra-nuclear genetic material in animals, exhibit strict maternal inheritance. Analyzing mtDNA sequences allows the determination of mitochondrial genetic information inherited by hybrid progeny, thus revealing their maternal parentage. Specific primers based on mtDNA effectively address the issue that hybrids contain nuclear genomes from both parents, which techniques like microsatellites cannot resolve [52]. In this study, specific primers were designed by screening single nucleotide polymorphism (SNP) loci based on differential sequence regions of Ai and Ap mtDNA [53]. The PCR method validated these primers, leading to the identification of Aip, Api, Aipi, Aipp, Apii, and Apip scallop strains. The results were consistent with those identified by Liu in the progeny of Ap and Ai crosses, confirming the maternal origin of the backcross population [54].

In conclusion, this study provides a deeper understanding of growth and development, gonadal development and differentiation, genetic diversity analyses and maternal parentage analyses in Aipi, Aipp, Apii, and Apip scallops. In particular, Aipp and Apip exhibit superior growth traits, and can serve as potential hybrid populations for subsequent germplasm improvement. Our research offers a theoretical basis for selecting scallop varieties with excellent traits and addressing the issue of inbreeding decline in scallop culture populations.

## 4. Materials and Methods

### 4.1. Brood Stock Collection

The Ai, Ap, and their hybrid progeny (Aip derived from Ai ♀ × Ap ♂, Api derived from Ap ♀ × Ai ♂) populations used in the experiments were sourced from the scallop culture area of Yangmadao, Yantai City, Shandong Province. They were transported at low temperatures to the scallop hatchery of Yantai Sea-Spring AquaSeeds Co., Ltd. in Laizhou, Shandong, China, in December 2021. The brood stocks of Aip and Api were produced from the 2020 hybridization and conservation efforts. Upon arrival, the scallops were placed in sand-filtered seawater for a fortnight to acclimate to the living environment, with the seawater temperature maintained at 10 ± 0.5 °C and salinity at 26 ± 1 ppt. After acclimatization, the seawater temperature was gradually increased to 20 °C at a rate of 0.5 °C per day to ensure simultaneous gonadal maturation in all four populations. During the temperature-raising period, feeding was performed four times a day. The feed consisted of *Chaetoceros* muelleri, *Isochrysis galbana*, and *Platymonas subcordiformis*, with a feeding concentration of approximately 5–8 × 104 cells/mL. Additionally, the seawater exchange was performed once each day during the morning and evening, with a seawater exchange volume of 50% of the total culture volume.

### 4.2. Spawning and Hybridization

Fifty individuals from Ai, Ap, Aip, and Api populations in good growth conditions with mature gonadal development were selected for the experiment. To induce spawning, scallops were exposed to air for 30 min and then placed in a storage pond with sand-filtered seawater at 24 °C. As soon as the scallops began to discharge sperm, they were immediately picked out and thoroughly rinsed with fresh water to inactivate sperm. Afterwards, each scallop was transferred individually into a beaker pre-filled with sand-filtered seawater at 24 °C. The status of each scallop in the beakers was continuously observed. When the parent scallops began spawning, the eggs were collected, and body surface and mantle were rinsed with sand-filtered seawater before being returned to the beaker to continue discharging. If no polar body was observed after 30 min of spawning (at 24 °C), the collected eggs were considered pure and were placed in a large beaker for reserve. Typically, scallops begin to discharge purer sperm after the peak of ovulation, and after 2–3 discharges, the semen can reach 99% purity. The sperm was filtered through a 500-mesh sieve and collected in a beaker. Eggs from Ai and the hybrid generation (Aip/Api) and sperm from Ai and Ap were collected using this method. Finally, separate fertilizations were performed to obtain purebred scallop populations (Ai) and backcrossed scallop populations (Aip ♀ × Ai ♂, denoted as Aipi; Aip ♀ × Ap ♂, denoted as Aipp; Api ♀ × Ai ♂, denoted as Apii; Api ♀ × Ap ♂, denoted as Apip). Three replicates were set up for each hybrid combination.

### 4.3. Early Embryonic Development, Larval and Juvenile Shellfish Cultivation

Before fertilization, egg density was determined by counting three replicate 1 mL aliquots under a light microscope, and sperm concentration was quantified using a hemocytometer. Fertilization was conducted by adding sperm to egg suspensions at a sperm-to-egg ratio of approximately 30:1. This ratio was optimized in preliminary experiments to achieve high fertilization rates while reducing the risk of polyspermy.

Sperm and eggs were gently mixed by swirling the beaker and incubated for fertilization for 30 min at 24 °C, after which the fertilization rate was assessed (each independent beaker is defined as one experimental unit). Fertilized eggs were filtered through a 500-mesh nylon screen and rinsed three times with 24 °C sand-filtered seawater. This washing procedure efficiently removes unbound spermatozoa whilst retaining the larger-diameter fertilized eggs. Following rinsing, the fertilized eggs were transferred to 100 L plastic buckets with continuous aeration for incubation at a stocking density of 10–15 eggs/mL. Seawater temperature was maintained at 24 °C, and salinity was kept at approximately 26 ppt. Seawater was not changed before fertilization, and the fertilized eggs were stirred every one to two hours. After fertilization, samples were taken from each fertilized egg group at regular intervals. The development of the hybrid embryos was continuously observed and photographed under a microscope to document their progression and the time required to reach each developmental stage.

When the embryos reached the D-larval stage, the hatch rate of each backcross combination was recorded, and healthy D-type larvae filtered through a 300-mesh sieve were transferred to 100 L plastic drums containing 24 °C sand-filtered seawater for larval cultivation, with continuous aeration. D-larvae were fed with golden algae (*Chrysophyceae*) at 50,000 to 10,000 cells/mL. In the later stages of larval development, additional feed such as C. muelleri and *P. subcordiformis* was provided as appropriate. The sand-filtered seawater was changed every 12 h, with half the water volume replaced each time.

When around 30% of the scallop larvae developed to the eye-point larval stage, attachment substances were placed in the buckets for larvae to settle on after metamorphosis. Following around 7 days of further cultivation after settlement, the larvae were transferred to shrimp ponds for nursery cultivation, with seedling bags replaced every three weeks. After 6 weeks of cultivation in the shrimp pond, the larvae were brushed off from the attachment substances, placed into bags with larger apertures according to their growth condition, and subsequently transferred to the open sea area of Yangmadao, Yantai, for aquaculture. Once the scallops exceeded 1.5 cm in shell height, they were placed in 10-layer lantern nets for adult grow-out in the sea. The density of scallops in the lantern nets was adjusted monthly to ensure sufficient growing space.

Samples were taken from the larvae of each backcross group and the parent group of Ai on the 5th, 10th, 15th, and 20th days of larval and juvenile shellfish development. The shell length growth was recorded through continuous observation and photographic measurements under a microscope. Additionally, 30 scallops were randomly selected during the monthly splitting period, and their growth and development indices, such as shell height, shell length, shell width, and wet weight, were measured and recorded.

Fertilization rate (%) = number of fertilized eggs/total number of eggs × 100%(1)

Hatching rate (%) = number of D − shaped larvae/number of fertilized eggs × 100%(2)

### 4.4. Histological Observations

During the growth and development stages, six scallops were randomly selected from each backcross combination every two weeks (6 individuals per group per time point). Intact gonadal tissues of these scallops were collected and fixed in 4% paraformaldehyde for over 24 h at 4 °C. The tissues were then prepared for paraffin sectioning through a process of graded alcohol dehydration (70%, 80%, 90%, 95%, and 100%), xylene transparency, and paraffin embedding. The dehydrated gonads were serially sectioned at 5 μm, dewaxed with xylene, rehydrated through gradient ethanol (100%, 95%, 80%, 70%), and finally stained with hematoxylin and eosin for observation. The development of gonadal tissue at various growth stages was examined under a microscope and documented with a high-resolution micro camera [55].

### 4.5. Measurement of Gonadal Differentiation-Related Gene Expression

RNA was extracted from Aipi, Aipp, Apii, and Apip tissues (including the coated membrane, gill, hepatopancreas, adductor muscle, gonadal male region, and gonadal female region) using FastPure Cell/Tissue Total RNA Isolation Kit V2 (Vazyme, Nanjing, China) and stored at -80 °C. RNA from the gonadal male and female regions was reverse-transcribed into cDNA for gene cloning using Evo M-MLV Plus 1st Strand cDNA Synthesis Kit (Accurate Biology, Hunan, China). Primers were designed using Primer Premier 5 based on the *Dmrt1* and *Foxl2* gene sequences obtained from the Ai transcriptome database and used to amplify these genes in the backcross populations Aipi, Aipp, Apii, and Apip. The *Dmrt1* gene was amplified using cDNA from the male region of the scallop gonad as a template, while the *Foxl2* gene was amplified using cDNA from the female region. PCR was conducted with 25 µL reaction mixtures containing 12.5 µL of 2 × Rapid Taq Master Mix, 8.5 µL of RNase-free water, 1 µL each of forward and reverse primers (10 µM), and 2 µL of the cDNA template. The cycling program was as follows: pre-denaturation at 95 °C for 5 min, followed by 35 cycles of denaturation at 95 °C for 30 s, annealing for 1 min, and extension at 72 °C for 1 min, with a final extension at 72 °C for 5 min. PCR products were detected using 1.2% agarose gel electrophoresis, recovered, purified using FastPure Gel DNA Extraction Mini Kit (Vazyme, Nanjing, China), and ligated with the pMD19-T vector (Takara, Kusatsu, Japan). The recombinant vector was transformed into *Escherichia coli* DH5α receptor cells (Takara, Kusatsu, Japan), which were then cultured. Positive clones were tested and sent for sequencing.

Reverse transcription was performed on RNA extracted from each tissue of the backcrossed scallops Aipi, Aipp, Apii, and Apip, yielding cDNA for each sample. Primer 5.0 software was used to design specific primers based on the sequences of *Dmrt1* and *Foxl2* genes in Aipi, Aipp, Apii, and Apip (Table S2). The elongation factor 1-alpha (EF-1α) served as an internal control, and SYBR Green II was used as the fluorescent dye. The expression of *Dmrt1* and *Foxl2* genes in different tissues of the backcross lines was detected using quantitative real-time fluorescence PCR (qRT-PCR) with three biological replicates (each from a distinct individual) and six technical replicates for each sample to minimize experimental errors. Relative expression was quantified by the 2^−ΔΔCt^ method [56]. Significant differences were determined using analysis of variance (ANOVA), where different superscript letters in each column indicate significant difference (*p*-value < 0.05).

### 4.6. Genetic Diversity Analyses

Genomic DNA was extracted from the adductor muscles of 120 adult scallops, which were derived from both purebred and backcrossed populations using the FastPure Blood/Cell/Tissue/Bacteria DNA Isolation Mini Kit-BOX 2 (Vazyme, Nanjing, China). Eleven SSR loci with high polymorphism and specificity were selected from bay scallops to compare the genetic diversity of parental and hybrid populations [57,58]. Primers of SSR loci used in this study were selected from published SSR primers from the bay scallops and shown in Table S3.

The PCR amplification process was the same as above. PCR products were separated by electrophoresis on a 9% non-denaturing polyacrylamide gel, stained with silver, and sized using DL2000 markers (Vazyme, Nanjing, China).

Genotypes of the six scallop cohorts were recorded from the gel images. The original “0” and “1” matrices were created through manual tape reading and analyzed using the PopGen32 software package. This analysis yielded the percentage of polymorphic loci (*P*), effective number of alleles (*E*), Nei’s gene diversity (*H*), and Shannon’s information index (*I*) among the groups. Collation and analysis of Nei’s genetic identity and genetic distance were performed based on the aforementioned data.

### 4.7. Statistical Analyses

Data on fertilization rate, hatching rate, and growth of the F2 generation backcross population were statistically analyzed using SPSS software (v26.0), with Ai growth serving as the control. All experiments were performed with at least three biological replicates, unless otherwise specified. For fertilization and hatching rate assays, each cross combination was set up in three independent beakers as biological replicates. To enhance the normality and homogeneity of the data, growth parameters were validated after logarithmic transformation with a base of ten. Differences in fertilization, hatching, and growth rates among the cohorts were analyzed using one-way ANOVA followed by Duncan’s tests [59]. Statistical significance was set at *p* < 0.05.

The shell length and wet weight of the backcrosses were used to calculate heterosis relative to bay scallops, using the following formulas.

(3) $$\mathrm{Heterosis},\;H(\%)=\frac{\bar{X}F2-\bar{X}Ai}{\bar{X}Ai}\times 100\%$$

(4) $$H_{\mathrm{Aipi}}\;(\%)=\frac{\bar{X}Aipi-\bar{X}Ai}{\bar{X}Ai}\times 100\%,$$

(5) $$H_{\mathrm{Aipp}}(\%)=\frac{\bar{X}Aipp-\bar{X}Ai}{\bar{X}Ai}\times 100\%,$$

(6) $$H_{\mathrm{Apii}}\;(\%)=\frac{\bar{X}Apii-\bar{X}Ai}{\bar{X}Ai}\times 100\%,$$

(7) $$H_{\mathrm{Apip}}(\%)=\frac{\bar{X}Apip-\bar{X}Ai}{\bar{X}Ai}\times 100\%,$$

where F2 is the shell length of the progeny of the backcross population, and *Ai* is the shell length of the bay scallop population. *H_Aipi_* (%), *H_Aipp_* (%), *H_Apii_* (%), and *H_Apip_* (%) denote the heterosis of the progeny of the respective backcross populations over the Ai parent.

### 4.8. Maternal Parentage Analyses

Adductor muscle tissues from scallops (Ai, Ap, Aip, Api, Aipi, Aipp, Apii, Apip) were sampled and stored at −80 °C after liquid nitrogen flash-freezing. Whole-genome DNA was extracted using FastPure Blood/Cell/Tissue/Bacteria DNA Isolation Mini Kit-BOX 2 (Vazyme, Nanjing, China) and stored at −20 °C. The mitochondrial DNA genome sequences of Ai and Ap were retrieved and downloaded from NCBI (sequence numbers KT161260.1 and KT161262.1) [54]. DNAMAN was employed for sequence comparison to identify mtDNA regions with significant differences between Ap and Ai (100–500 bp is optimal). Based on the specific mtDNA regions, primers were designed and used for PCR amplification with genomic DNA of Ai and Ap (Table S1). The sequencing process of mtDNA fragments was conducted in the same way as the sequencing of *Foxl2* and *Dmrt1* above. Subsequently, the sequencing results were compared with the full sequence of the Ap mitochondrial genome to confirm that the DNA fragments obtained by PCR amplification originated from Ap mtDNA templates. Finally, 20 samples each of Aip, Api, Aipi, Aipp, Apii, and Apip were randomly selected and amplified by PCR using the screened specific primers. The products were detected via electrophoresis on a 1.2% agarose gel, completing the identification of the maternal parentage of Aip, Api, Aipi, Aipp, Apii, and Apip.

## Figures and Tables

**Table 1:** The development time of Ai, Aipi, Aipp, Apii, and Apip at embryonic and larval stages.

Cohorts	Ai	Aipi	Aipp	Apii	Apip
First polar body	9.7 ± 1.53 min ^c^	12.3 ± 1.31 min ^b^	15.1 ± 1.27 min ^a^	12.7 ± 1.13 min ^ab^	14.7 ± 1.59 min ^ab^
Secondary polar body	14.3 ± 1.15 min ^c^	20.9 ± 1.26 min ^a^	20.2 ± 1.31 min ^ab^	18.6 ± 1.45 min ^b^	19.5 ± 1.11 min ^ab^
Two-cell	40.3 ± 2.52 min ^c^	58.6 ± 3.09 min ^a^	60.2 ± 5.21 min ^a^	49.6 ± 2.58 min ^b^	55.2 ± 2.51 min ^ab^
Four-cell	0.8 ± 0.05 h ^b^	1.5 ± 0.06 h ^a^	1.5 ± 0.11 h ^a^	1.4 ± 0.09 h ^a^	1.4 ± 0.07 h ^a^
Eight-cell	1.5 ± 0.04 h ^b^	2.0 ± 0.08 h ^a^	2.1 ± 0.12 h ^a^	2.0 ± 0.10 h ^a^	2.0 ± 0.11 h ^a^
Blastula	5.0 ± 0.11 h ^d^	6.0 ± 0.15 h ^b^	5.9 ± 0.13 h ^bc^	5.7 ± 0.21 h ^c^	6.3 ± 0.15 h ^a^
Gastrula	11.0 ± 0.24 h ^c^	12.0 ± 0.23 h ^b^	12.4 ± 0.17 h ^a^	12.2 ± 0.13 h ^ab^	12.1 ± 0.14 h ^ab^
Trochophore	17.1 ± 0.28 h ^a^	17.9 ± 0.26 h ^a^	18.0 ± 1.33 h ^a^	18.1 ± 1.52 h ^a^	18.3 ± 1.21 h ^a^
D-larvae	20.9 ± 0.29 h ^d^	41.4 ± 0.31 h ^b^	40.1 ± 0.24 h ^c^	42.2 ± 0.19 h ^a^	41.7 ± 0.86 h ^ab^
Umbo larvae	6.2 ± 0.18 d ^d^	7.0 ± 0.13 d ^c^	8.0 ± 0.11 d ^a^	7.2 ± 0.21 d ^c^	7.7 ± 0.2 d ^b^
Eyed larvae	9.1 ± 0.30 d ^c^	12.0 ± 0.23 d ^a^	11.3 ± 0.22 d ^b^	11.1 ± 0.36 d ^b^	12.1 ± 0.19 d ^a^
Juveniles	12.7 ± 0.48 d ^b^	16.0 ± 0.35 d ^a^	16.1 ± 0.25 d ^a^	16.1 ± 0.27 d ^a^	15.9 ± 0.22 d ^a^

Data are presented as X ± SD (mean ± standard deviation) Values that lack a single conserved letter differ significantly (*p* < 0.05) from each other.

**Table 2:** Growth index and their heterosis of Ai, Aipi, Aipp, Apii, and Apip during the grow-out stage.

Cohorts	Day 60	Day 90	Day 120		Day 150	
	Shell Length (mm)	Shell Length (mm)	Shell Length (mm)	Wet Weight (g)	Shell Length (mm)	Wet Weight (g)
Ai	1.7 ± 0.07 ^c^	12.7 ± 0.93 ^c^	32.2 ± 2.29 ^b^	5.3 ± 1.27 ^c^	42.5 ± 2.77 ^b^	14.1 ± 2.92 ^d^
Aipi	6.2 ± 0.13 ^a^	19.2 ± 2.61 ^ab^	35.2 ± 4.27 ^b^	7.3 ± 2.59 ^c^	44.8 ± 2.39 ^b^	18.2 ± 2.9 ^c^
Aipp	5.5 ± 1.21 ^a^	23.4 ± 2.54 ^a^	40.9 ± 5.04 ^a^	13.1 ± 3.90 ^a^	54.4 ± 4.18 ^a^	30.8 ± 6.39 ^a^
Apii	5.1 ± 0.61 ^a^	20.7 ± 2.56 ^ab^	39.4 ± 3.63 ^a^	11.7 ± 2.61 ^ab^	52.5 ± 3.99 ^a^	24.0 ± 4.51 ^b^
Apip	3.7 ± 0.26 ^b^	18.8 ± 2.71 ^b^	39.4 ± 4.31 ^a^	11.1 ± 3.17 ^ab^	52.9 ± 4.05 ^a^	28.1 ± 4.77 ^a^
*H_Aipi_* (%)	264.12	51.42	9.31	37.17	5.48	29.08
*H_Aipp_* (%)	222.94	84.76	26.75	147.92	27.98	118.44
*H_Apii_* (%)	201.76	63.43	22.37	121.32	22.75	70.28
*H_Apip_* (%)	118.24	48.58	22.12	109.81	24.54	99.50

*H_Aipi_* (%), *H_Aipp_* (%), *H_Apii_* (%), and *H_Apip_* (%) indicate the heterosis of Aipi, Aipp, Apii, and Apip over the Ai. Data are presented as X ± SD (mean ± standard deviation). The sample size was 30. Values that lack a single conserved letter differ significantly (*p* < 0.05) from each other.

**Table 3:** Genetic diversity parameters of Ai, Ap, Aipp, Aipi, Apii, and Apip.

Species	P	Na	Ne	H	I
Ai	29.85	1.2985	1.2498	0.1351	0.1924
Ap	25.37	1.2537	1.2074	0.1095	0.1563
Aipp	68.66	1.6866	1.5295	0.2919	0.4205
Aipi	61.19	1.6119	1.4346	0.2460	0.3595
Apii	71.64	1.7164	1.5148	0.2904	0.4231
Apip	67.16	1.6716	1.4869	0.2691	0.3896

P: Percentage of polymorphic loci; Na: Observed number of alleles; Ne: Effective number of alleles; H: Nei’s gene diversity; I: Shannon’s Information index.

**Table 4:** Nei’s genetic identity and genetic distance of Ai, Ap, Aipp, Aipi, Apii, and Apip.

Species	Ai	Ap	Aipp	Aipi	Apii	Apip
Ai	-	0.5268	0.6268	0.7415	0.7124	0.5707
Ap	0.6408	-	0.6441	0.6060	0.6199	0.6929
Aipp	0.3809	0.3374	-	0.7034	0.7705	0.6852
Aipi	0.2991	0.4712	0.3044	-	0.7927	0.7369
Apii	0.3392	0.4782	0.2607	0.2023	-	0.7742
Apip	0.4327	0.3669	0.3393	0.3053	0.2559	-

Values above diagonal indicate Nei’s genetic identity and those below diagonal indicate genetic distance.

## Data Availability

Data will be made available on request.

## Supplementary Materials

The following supporting information can be downloaded at: https://www.mdpi.com/article/10.3390/ijms27167290/s1.

## Abbreviations

The following abbreviations are used in this manuscript:

Ai	*Argopecten irradians*
Ap	*Argopecten purpuratus*
Aip	F1 hybrid from Ai ♀ × Ap ♂
Api	F1 hybrid from Ap ♀ × Ai ♂
Aipi	backcross from Aip ♀ × Ai ♂
Aipp	backcross from Aip ♀ × Ap ♂
Apii	backcross from Api ♀ × Ai ♂
Apip	backcross from Api ♀ × Ap ♂
cDNA	complementary DNA
COI	cytochrome c oxidase subunit I
EF-1α	elongation factor 1-alpha
eQTL	expression quantitative trait locus
mtDNA	Mitochondrial DNA
ND	NADH dehydrogenase
qRT-PCR	quantitative real-time polymerase chain reaction
SSRs	Simple Sequence Repeats
SNP	single nucleotide polymorphism
