# Supplementary material for: Deciphering Production Potential and Gonadal Differentiation of the Backcross Hybrids Between Argopecten irradians and A. purpuratus
Source: Int J Mol Sci. 2026 Aug 15;27(16):7290. doi: 10.3390/ijms27167290 (PMC13513516; doi:10.3390/ijms27167290)
Supplement: Supplementary file 1 [file ijms-27-07290-s001.zip › ijms-4477049-supplementary.pdf]

Figure legends

Figure. S1 Sex phenotypes and gonadal development in Aipi, Aipp, Apii and Apip. The original histological images.

Figure. S2 Full electropherograms of amplification products using Specific primers.

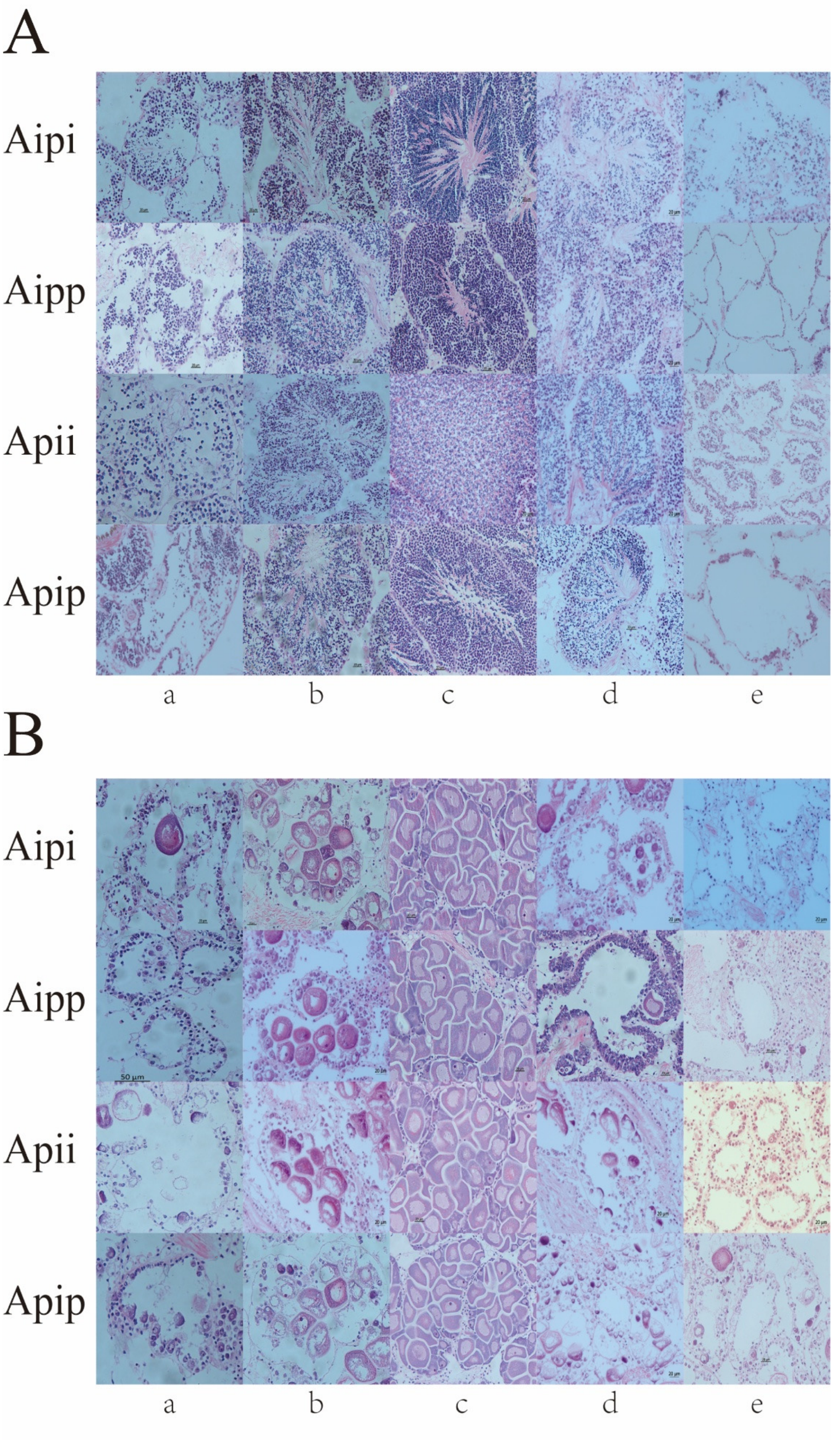

Figure S1.

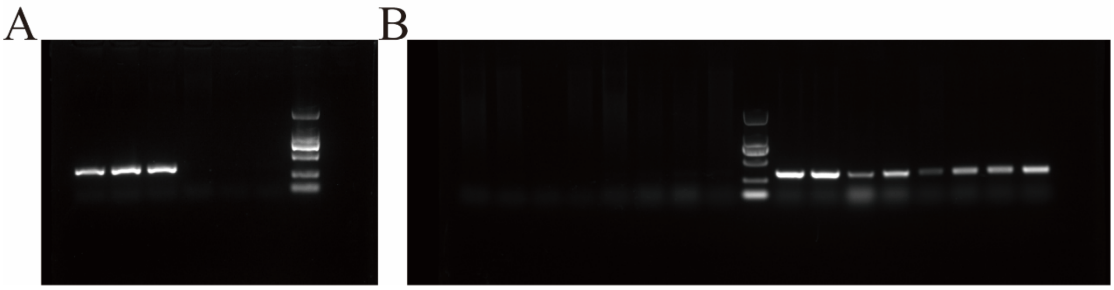

Figure S2.

Table Legends

Table S1. Primer sequences for the maternal parentage analyses

Table S2. Primer sequences of *Dmrt1* gene *Foxl2* gene and reference gene for qRT-PCR

Table S3. Primer sequences for the genetic diversity analysis

Table S1 Primer sequences for the maternal parentage analyses

Primer	Primer Sequence	annealing temperature (℃)
mbqq-1	F: TAGAAATTTCACTCTTATATACTC<br>R: TTCTGTACCTGAAATCTAGC	56
mbqq-2	F: TTGGCGTCTAGCTCACCCC<br>R: GGGGCTACGACTAAATTCCTGT	58
mbqq-3	F: GCGAGGCTAATCTTCGGGG<br>R: TCCTCATAATCGTAGTAACCCC	57
mbqq-4	F: CAGGGCTGAAGAGACAAAGG<br>R: AAAATTTCGATGATTCCCCC	54
mbqq-5	F: AGACCCGGGATAAAGGCTAA<br>R: ACAATCGAATCCTGAGCCAC	55
mbqq-6	F: AAAGGTTTGGAAAAGTTGTGGG<br>R: TAATCGTAGTAACCCCTCCC	53
mbqq-7	F: GGTTTGGAAAAGTTGTGGGG<br>R: GCCTAAAAACAACCCTCACC	53
mbqq-8	F: AGGTTTGGAAAAGTTGTGGG<br>R: GGCCTAGACAGCCTAAAAACAA	54
mbqq-9	F: ATACCTCGCCGCATCCCT<br>R: CGCAATGCCATAACCTCTGT	57
mbqq-10	F: TATGAGGTGTCCCCCAAGTC<br>R: ACTGGCAGACAAACAAATCGT	55

Table S2 Primer sequences of *Dmrt1* gene *Foxl2* gene and reference gene for qRT-PCR

Primer	Primer Sequence	Fragment length (bp)
Aipi-qDmrt1	F: GTTCGGTAGAGTCGCTGATTG<br>R: GCTCTCCTCCTCATCCTTCATA	128
Aipp-qDmrt1	F: CAGATTCCCTACAGATCCTCATTC<br>R: CTATGGCTTTCTGGGTGTCTC	102
Apii-qDmrt1	F: CAGATTCCCTACAGATCCTCATTC<br>R: GACAGCGTCAATCCTCTCTATG	119
Apip-qDmrt1	F: GGAAACAAGAGGAGGATATGGG<br>R: CCAATTTGTCGACGTTGTAGTG	105
Aipi-qFoxl2	F: AGTTTCGACTCTTTCGTGGAG<br>R: CACTTCCAACATGCGGTTTC	102
Aipp-qFoxl2	F: TGCAGGAAGTTTGAGGAAGAC<br>R: CTTGTCGCCTGATTCTTTGATTG	108
Apii-qFoxl2	F: ACGGAAGACCTGAAACCAATTA<br>R: CTTCCTCAAACTTCCTGCATTTAG	103
Apip-qFoxl2	F: AGTTTCGACTCTTTCGTGGAG<br>R: CACTTCCAACATGCGGTTTC	102
ef-1α	F: CCTTTGCCCCATCTCAACTGTC<br>R: CAGCAACGTTTCCTCTCTTCAATTC	144

Table S3 Primer sequences for the genetic diversity analysis

Primer	Primer Sequence	annealing temperature (℃)
ssr-1	F: AGACAACCGAGGAACAATG<br>R: TTAGTGGATCAGATAGTCAAAGC	56
ssr-2	F: ACTGAAGGCACTATGTTTTGAC<br>R: ACAAGCCTGCAGATATTTCC	59
ssr-3	F: AAGATGCAAATTGCTGTCAGG<br>R: CATTCACATCCACCCATACAAG	55
ssr-4	F: TATACGCTAAACCATCGCYG<br>R: AAAAGTTACGAAACACTATCAAAAG	58
ssr-5	F: AGGCATTGAAGCAGAGGCTGAC<br>R: CATGTCATCATCCGACTCCTCG	61
ssr-6	F: TTAAAACCAATGGTCAACTAGAAC<br>R: GAAAATATGATAACGCATGTGC	58
ssr-7	F: ACATCTCCTGATCTGAATGACC<br>R: GAGGGTAGGAATGGGGATAG	58
ssr-8	F: AAACAGAAACAATAGAGACAGTG<br>R: CTAGTATCTTTTGGAAACTACAGT	55
ssr-9	F: GCACAAATAGAATCAGTAACCAGAA<br>R: CATTTGACAGCGGTGGAGC	58
ssr-10	F: TTCTGCTCATAGACATTTTCC<br>R: ATAAAGATTAACGATATTTCATTG	52
ssr-11	F: ATACCATTTCCATTATGACG<br>R: ATGAACAATGTGCTTGAGG	54
